# Tg in Adults as a Sensitive Biomarker of Iodine Status: A 5-Year Follow up Population Study in Different Levels of Iodine Intake Regions

**DOI:** 10.1371/journal.pone.0135553

**Published:** 2015-08-12

**Authors:** Wei Chong, Xiaoguang Shi, Zhongyan Shan, Xiaochun Teng, Di Teng, Haixia Guan, Yushu Li, Ying Jin, Xiaohui Yu, Chenling Fan, Fan Yang, Hong Dai, Yang Yu, Jia Li, Yanyan Chen, Dong Zhao, Fengnan Hu, Jinyuan Mao, Xiaolan Gu, Rong Yang, Yajie Tong, Weibo Wang, Tianshu Gao, Chenyang Li, Weiping Teng

**Affiliations:** 1 Department of Emergency, The First Affiliated Hospital of China Medical University, Shenyang, Liaoning Province, People’s Republic of China; 2 Department of Endocrinology and Metabolism, Institute of Endocrinology, Liaoning Provincial Key Laboratory of Endocrine Diseases, The First Affiliated Hospital of China Medical University, Shenyang, Liaoning Province, People’s Republic of China; University of Munich, GERMANY

## Abstract

This study was to evaluate the usefulness of serum thymoglobulin (Tg) in adults to assess iodine status through a 5-year cohort study which was conducted in three regions with different levels of iodine intake: mild deficiency, more than adequate, and excess, from 1999 to 2004 in China. A total of 3099 subjects over 14 years old with normal serum levels of Tg in 1999 were eligible, of whom 2448 were followed in 2004. Serum levels of thyroid hormones and thyroid autoantibodies as well as urine iodine were measured, and B-mode ultrasonography of the thyroid was performed. A general linear model was performed to determine the determinant factors of serum Tg. Among subjects with mildly deficient iodine intake, those with more than adequate intake, and those with excessive intake, the baseline levels of serum Tg were substantially different (7.5μg/L, 5.9μg/L, and 6.8μg/L respectively, *P*<0.01), which were associated with age, sex, the rate of positive TgAb, abnormal thyroid volume, abnormal TSH, and positive personal history of thyroid diseases. The data from 1856 subjects with normal range of thyroid parameters but no personal history of thyroid diseases were analyzed to clarify the effect of iodine intake on Tg. Among these three regions, the serum Tg levels were substantially different in both 1999 and 2004, with a similar pattern for increased Tg (ΔTg) (3.1μg/L, 2.5μg/L and 3.5μg/L respectively, *P*<0.01). The general linear model analysis revealed that age, Tg, and baseline TSH levels were the determinants of ΔTg besides iodine intake. In conclusion, serum Tg in adults, resulting from a time-accumulative effect of iodine exposure, is a useful biomarker of regional iodine intake.

## Introduction

As a basic component of thyroid hormones, the level of iodine in human body should maintain within a safe range, because either deficiency or excess is harmful to thyroid. Unfortunately, the latest global data suggested that, in 2011, iodine intake was inadequate in 32 countries, whereas more than adequate or even excessive iodine intake occurred in 47 countries [1]. Therefore, it is necessary to improve the monitoring procedures of iodine levels in populations while controlling iodine deficiency disorders (IDDs).

Four sensitive indicators have been recommended for the assessment of iodine nutrition: urinary iodine concentration, goiter rate, serum TSH, and Tg [2]. Most iodine absorbed in the body eventually appears in the urine. Therefore, urinary iodine excretion is a good marker of very recent dietary iodine intake. In individuals, urinary iodine excretion can vary somewhat from day to day and even within a given day. This variation tends to even out among populations and the median urinary iodine concentration is the indicator most commonly assessed to reflect population iodine status [3]. However, urinary iodine might underestimate iodine deficiency of gravida due to increasing nephric iodine clearance [4]. Goiter rate reflects long-term iodine nutrition (from months to years). However, thyroid size decreases at a considerably slow rate after iodine repletion and goiter in children is a poor IDD indicator even after several years of iodized salt consumption [5]. Serum TSH is a reliable indicator of iodine nutrition especially for the newborn, but not for the others [3]. Compared with all above three indicators, serum Tg reflects not only thyroid function but also recent iodine intake (from weeks to months). Its major determinants are thyroid cell mass and TSH stimulation when the thyroid has not been damaged [6], which suggests that serum Tg level is able to indicate the hyper-stimulation of TSH and thyroid hyperplasia [3]. In addition, serum Tg concentration was reported to remain at similar levels during pregnancy and after delivery [7]. Thus, serum Tg has been proposed as a potential IDD indicator and might have particular values in detecting short-term changes of thyroid function in response to salt iodization [8].

Most of the studies in children between age 5 and 14 appear to support the use of Tg to assess iodine status in this age group, as proposed by Zimmermann [9]. One cross-sectional study in China conducted among schoolchildren of three different iodine intake regions suggested that serum Tg values in excessive iodine intake region were markedly higher than those in the mild iodine deficient region and more than adequate iodine region [10]. For adults, a cross-sectional study conducted in regions of mild and moderate iodine deficiency in Denmark found a negative correlation between the serum Tg level and the level of urinary iodine [11]. One prospective study performed in the same regions, however, demonstrated a higher efficacy of Tg than that of thyroid volume, indicating a difference between pre- and post-iodization values [12]. Up to date, the influence of excessive iodine intake on serum Tg in adults remains unclear.

We previously conducted a cross-sectional study and demonstrated that serum Tg level was affected by gender, amount of iodine intake, serum TSH level, and thyroid volume, based on the data collected from a five-year follow-up population survey in adults from both deficient and more than adequate or excessive iodine intake regions, known as “iodine induced thyroid disease (IITD) program” from 1999 to 2004 [13]. In the present study, we aim to analyze the temporal changes of serum Tg levels during the follow-up period, and to confirm the hypothesis that Tg in adults could be a sensitive marker for monitoring regional iodine exposure.

## Subjects and Methods

### Ethics statement

The research protocol and informed consent form were approved by the Committee on Human Experimentation of China Medical University. Research protocols were carefully explained to all voluntary participants. When minors were enrolled in our study, the research protocols were carefully explained to their parents. Written informed consent regarding conduct of the survey was obtained from all adult participants or the parents of minor participants. We protected the privacy of individuals in processing personal data and maintained confidentiality of individual records and accounts.

### Study regions and Subjects

Thirteen villages with similar age, gender composition, economical status, and cultural and medical conditions were selected for the study, from 3 different iodine intake regions in China: 4 villages in Panshan, 7 villages in Zhangwu, and 2 villages in Huanghua. The inhabitants in Panshan traditionally consumed locally produced salt (less than 3.4 mg/kg iodine) and thus had a long-term mild deficiency in iodine intake. Residents of Zhangwu had been mildly deficient in iodine intake before 1995, and the intake increased after salt iodization instituted nationwide in 1996, so that the iodine intake had been more than adequate since then. Huanghua is a region in which residents have excessive iodine intake owing to the high iodine content in the drinking water (96 to 228 μg/L). Median urinary iodine of school children in Panshan, Zhangwu, and Huanghua counties were 84 μg/L, 243 μg/L, and 651 μg/L respectively in 1999, and 88 μg/L,214 μg/L, and 634 μg/L in 2004, indicating no substantial difference of the iodine nutritional status over the 5-year period in each study region [14]. According to the epidemiological criteria for assessing iodine nutrition based on median urinary iodine concentrations of school-age children, the iodine status of children in these three regions from 1999 to 2004 was considered as mild deficient, more than adequate, and excessive respectively [3]. The inhabitants who were Han Nationality, were over 14 years old, had lived in these regions for more than 10 years, and had serum Tg levels in the reference range, composed our study population. A total of 3009 individuals were enrolled in 1999. The enrollment included answering questionnaires, undergoing thyroid ultrasonography, and taking relevant venous blood tests. In 2004, 2448 of the original enrollees were followed with a response rate of 81.4%.

### Measurement of laboratory parameters

The chemiluminescence immunoassay was performed to measure serum Tg, TgAb, TPOAb, and TSH levels (Diagnostic Products Corporation, Los Angeles, CA,), as previously described [14]. To ensure the compatibility of data, the tests were performed in a masked manner by the same group of technicians, using the same equipment, in both 1999 and 2004. The normal range of serum Tg, TgAb, TPOAb and TSH were 0.87–67.1 ng/mL [13], <40 IU/mL, <50 IU/mL and 0.3–4.8 mU/L respectively [14].

### Ultrasonography examination of thyroid volume (TV)

Thyroid ultrasonography was also performed [15] by trained professionals using the same equipment (model SA600 with 7.5-MHz linear transducers, Medsion) in both performances. Goiter was defined as a thyroid volume exceeding 19.4 ml for women and 25.6 ml for men. This definition was derived from the mean±2SD thyroid volume obtained in 392 individuals from one region (median urinary iodine, 126 μg/L [25th to 75th percentile, 112μg/L to 188μg/L]), who did not have thyroid diseases, family histories of thyroid disease, detectable antithyroid antibodies, or ultrasonographically evident goiter/thyroid nodules [14].

### Statistical analysis

The difference among the three regions was tested with *chi-square test* for categorical variables, *ANOVA* for continuous variables with normal distribution, and *nonparametric rank test* for continuous variables without normal distribution. The distributions of serum Tg and ΔTg (5-year change of serum Tg) in categorical variables were examined with *nonparametric rank test*, and their correlations with continuous variables were tested with *Spearman correlation*. The general linear model was performed to identify the determinant factors of serum Tg. Items with *p*>0.15 were eliminated one at a time in the sequence of *p* values. When an item was eliminated, if the change of any remaining parameter was greater than 20%, this item would be kept in the model as a confounder, and the interaction between the confounder and the affected item would be examined. In this study, no confounder was found during elimination, and therefore, the interaction between confounders was not found. Besides, we also performed spearman correlation to clarify the collinearity among all continuous variables before multivariate analysis. In this study, no collinearity data was found because of the low correlation among the continuous variables, ranging from 0.01 to 0.16 (lower than 0.6).

The continuous variables with normal distribution and without normal distribution and categorical variables were expressed by the *mean* (standard deviation), the *median* (inter-quartile range) and the number (%) respectively. *SAS* for Windows, version 8.2, was used for all statistical analysis.

## Results

### Serum levels of Tg and subject characteristics in three iodine intake regions at baseline

The median serum Tg levels in the mild deficient iodine region [Panshan: 7.5(4.4~13.1) ng/mL]) and the excessive iodine region [Huanghua: 6.8(3.6~11.2) ng/mL] were much higher than that in the more than adequate iodine region [Zhuangwu: 5.9(3.2~10.7) ng/mL] at baseline (1999), *p*<0.05. Among mild deficient, more than adequate, and excessive iodine regions, the difference in age [35.7(11.9) years, 39.5(13.1) years, and 36.6(12.6) years] and goiter rate [142(23.2%), 187(17.3%), and 45(6.0%)] were substantial (*p*<0.0001), but the distributions of gender, the presence of abnormal TSH, positive TPOAb, positive TgAb, and the status of family history/personal histories of thyroid were not substantially different (Table 1).

**Table 1:** Serum level of Tg and subjects’ characteristics of three iodine intake regions at baseline.

Factors	Iodine intake status of regions			*p*
	Panshan: Mild deficient (n = 613)	Zhanwu: More Than Adequate (n = 1084)	Huanghua: Excessive (n = 751)	
Tg (ng/mL)[Median(interquartile range)]	7.5(4.4~13.1)^a^	5.9(3.2~10.7) ^a^	6.8(3.6~11.2) ^a^	<0.0001
Age(years)[Mean(standard deviation)]	35.7(11.9) ^a^	39.5(13.1) ^a^	36.6(12.6) ^a^	<0.0001
Sex [n (%)]				
Male	132(21.5)	256(23.6)	177(23.6)	
Female	481(78.5)	828(76.4)	574(76.4)	0.5762
TSH [n (%)]				
Normal	574(93.6)	979(90.4)	685(91.2)	
Abnormal	39(6.4)	104(9.6)	66(8.8)	0.0690
TPOAb [n (%)]				
Negative	564(92.0)	993(91.6)	690(91.9)	
Positive	49(8.0)	91(8.4)	61(8.1)	0.9537
TgAb [n (%)]				
Negative	574(93.6)	1019(94.1)	711(94.7)	
Positive	39(6.4)	64(5.9)	40(5.3)	0.7141
Goiter [n (%)]				
Absent	471(76.8)	897(82.7)	706(94.0)	
Present	142(23.2) ^a^	187(17.3) ^a^	45(6.0) ^a^	<0.0001
Family histories of thyroid diseases [n (%)]				
Absent	562(92.0)	992(91.8)	682(91.2)	
Present	49(8.0)	89(8.2)	66(8.8)	0.8501
Personal histories of thyroid diseases [n (%)]				
Absent	590(96.4)	1047(96.8)	720(96.1)	
Present	22(3.6)	35(3.2)	29(3.9)	0.7625

^a^: Compared every two groups *p*<0.05.

### Univariate analysis of factors in relation to serum Tg levels at baseline (except for the factor of region)

The serum Tg levels were significantly higher (*p*<0.0001) in females [6.8(3.7~12.6) ng/mL vs. males 5.7(3.3~9.4) ng/mL], those with abnormal TSH [9.0(4.0~20.1 ng/mL) vs. normal TSH 6.4(3.6~10.9) ng/mL], negative TgAb [6.8(3.7~11.9) ng/mL vs. positive TgAb 3.1(1.4~6.3) ng/mL], and goiter [7.7(3.7~18.4) ng/mL vs. no goiter 6.4(3.6~10.9) ng/mL]. Age was positively related to serum Tg level. The effects of TPOAb and family history of thyroid diseases on serum Tg level were not statistically substantial, whereas personal history of thyroid diseases was weakly related to serum Tg level (*p* = 0.07) (Table 2).

**Table 2:** Univariate analysis of other factors except for in relation to serum Tg at baseline.

Factors	Tg (ng/mL)		*p*
	n	Median(interquartile range)	
Sex			
Male	565	5.7(3.3~9.4)	
Female	1883	6.8(3.7~12.6)	<0.0001
TSH			
Normal	2238	6.4(3.6~10.9)	
Abnormal	209	9.0(4.0~20.1)	<0.0001
TPOAb			
Negative	2247	6.6(3.7~11.5)	
Positive	201	5.7(2.4~14.1)	0.1213
TgAb			
Negative	2304	6.8(3.7~11.9)	
Positive	143	3.1(1.4~6.3)	<0.0001
Goiter			
Absent	2074	6.4(3.6~10.9)	
Present	374	7.7(3.7~18.4)	<0.0001
Family histories of thyroid diseases			
Absent	2236	6.6(3.6~11.6)	
Present	204	6.5(3.5~10.9)	0.4826
Personal histories of thyroid diseases			
Absent	2357	6.5(3.6~11.3)	
Present	86	8.2(2.8~20.7)	0.0717
*Spearman correlation (r)*			
Age (years)	2448	0.06	0.0046

### Multivariate analysis of risk factors to serum Tg level at baseline

According to the result of the general linear model analysis, the difference of iodine intake was substantially associated with the serum Tg level. Serum Tg level in Panshan (mild iodine deficiency: *β* = 0.09, *p*<0.001) and Huanghua (excessive iodine: *β* = 0.04, *p* = 0.0711) were much higher than that in Zhangwu (more than adequate iodine). Except for the iodine intake, age (*β* = 0.06, *p* = 0.0035), gender (B = -2.34, *p*<0.0001), positive TgAb (*β* = 0.17, *p*<0.05), goiter (*β* = -0.16, *p*<0.0001), abnormal TSH (*β* = -0.15, *p*<0.0001), and personal history of thyroid diseases (*β* = -0.06, *p* = 0.0070) were all associated with the serum Tg level.

### The characteristics of the cohort population and the changes of serum Tg level during the follow-up

In order to exclude the confounding effects, the data from a selected subgroup consisting of 1856 subjects who had negative TgAb, normal TV, and normal TSH, and negative personal history of thyroid diseases were chosen to clarify the determinant effect of iodine intake on serum Tg. The serum levels of Tg among mild deficient, more than adequate, and excessive iodine regions were substantially different in both 1999 [7.8(4.5~13.2) ng/mL, 5.6(3.2~9.4) ng/mL and 6.6 (3.7~10.5) ng/mL] and 2004 [11.2(6.2~19.9) ng/mL 8.6(4.7~14.5) ng/mL and 9.7(5.7~ 18.9) ng/mL], respectively, *p*<0.0001. In particular, compared with that in the more than adequate iodine region, the median serum levels of Tg in both mild deficient and excessive iodine regions were substantially higher (*p*<0.05). The median serum levels of Tg were substantially higher in 2004 than those in 1999 in all regions (*p*<0.01). In addition, the change of serum Tg (ΔTg) was substantially different among three regions [3.1(-0.2~8.0) ng/mL, 2.5(0.3~ 6.1) ng/mL and 3.5(0.7~ 9.0) ng/mL, *p* = 0.0021]. (Table 3).

**Table 3:** Subjects characteristics and the 5-year changes of serum Tg (ΔTg) in three regions (n = 1856).

	Panshan: Mild deficient (n = 613)	Zhanwu: More Than Adequate (n = 1084)	Huanghua: Excessive (n = 751)	*p*
1999 Tg (ng/mL) [Median(interquartile range)]	7.8(4.5~13.2) ^a^	5.6(3.2~9.4)	6.6 (3.7~10.5) ^a^	<0.0001
2004 Tg (ng/mL) [Median(interquartile range)]	11.2(6.2~19.9) ^a^	8.6(4.7~14.5)	9.7(5.7~ 18.9) ^a^	<0.0001
ΔTg (ng/mL) [Median(interquartile range)]	3.1(-0.2~8.0)^b^	2.5(0.3~ 6.1)	3.5(0.7~ 9.0) ^a^	0.0021
Age(Age) [Mean(standard deviation)]	36.0(11.9) ^a^	39.5(12.3)	37.1(12.0) ^a^	<0.0001
Sex [n (%)]				
Male	98(22.7)	214(27.1)	163(25.8)	
Female	334(77.3)	577(72.9)	470(74.2)	0.2449
TSH [n (%)]				
Normal	424(98.1)	758(95.8)	614(97.0)	
Abnormal	8(1.9)	33(4.2)	19(3.0)	0.0832
TgAb [n (%)]				
Negative	415(96.1)	770(97.3)	607(95.9)	
Positive	17(3.9)	21(2.7)	26(4.1)	0.2686
Goiter [n (%)]				
Absent	422(97.7)	776(98.1)	616(97.3)	
Present	10(2.3)	15(1.9)	17(2.7)	0.6074

^a^: Compared with Zhangwu *p*<0.05;

^b^: Compared with Zhangwu *p*<0.15.

The population characteristics of the three iodine intake regions in 2004 in this cohort were shown in Table 3. There was no substantial difference in the incidence of abnormal TSH, positive TgAb, or goiter among the three regions.

### Univariate analysis of the factors (except for the geographic difference) in relation to ΔTg in the selected cohort population

Even though the baseline levels of the Tg and TSH were in the normal ranges, they were substantially related to the ΔTg (*p* = 0.0047 and 0.0478 respectively). In addition, ΔTg was substantially dependent on age at baseline, *p*<0.0001. However, gender and baseline levels of TgAb and TV did not substantially contribute to ΔTg (Table 4).

**Table 4:** Univariate analysis of other factors in relation to ΔTg (except the factor of area).

Factors	ΔTg (ng/mL)		*p*
	n	Median(interquartile range)	
Sex			
Male	475	2.8 (0.7~6.4)	
Female	1381	3.0 (0.2~ 7.8)	0.8819
*Spearman correlation (r)*			
Age (year)	1856	0.10	<0.0001
Tg (ng/mL) (1999)	1856	0.07	0.0047
TSH (mU/L) (1999)	1856	0.05	0.0478
TgAb (IU/mL) (1999)	1856	-0.02	0.2849
TV (ml) (1999)	1856	1.66×10^−3^	0.9430

### Multivariate analysis of the determinant factors of ΔTg in the selected cohort population

The results of the general linear model analysis demonstrated that different iodine intake had a substantial effect on ΔTg. ΔTg values in Panshan (mild iodine deficiency: *β* = 0.04, *p* = 0.1188) and Huanghua (excessive iodine: *β* = 0.05, *p* = 0.0369) were much higher than that in Zhangwu (more than adequate iodine). In addition, the baseline level of Tg (*β* = 0.09, *p* = 0.0001), TSH (*β* = 0.07, *p* = 0.0032), and age (B = 0.07, *p* = 0.0296) were found to be the independent determinant factors of ΔTg. Furthermore, the contribution of regions and age to the model R-Square accounted for 11.7% and 7.8%, respectively, which suggested iodine intake affected ΔTg more than age did (Table 5).

**Table 5:** The general linear model analysis for clarifying the determinant factors of ΔTg level of the selected cohort population.

Variables	Parameter Estimate (B)			Standardized Estimate (β)	Contribution to the model R-Square (%)
		95%CI	*p*		
Intercept	-1.44	-4.70–1.81	0.3842		
Sex(male vs. female)^a^	-1.03	-2.80–0.75	0.2559	-0.03	
Age (year)	0.07	0.01–0.13	0.0296	0.05	7.84
Tg (ng/mL) (1999)	0.20	0.10–0.30	0.0001	0.09	45.72
TSH (mU/L) (1999)	1.30	0.43–2.16	0.0032	0.07	26.17
Region					11.72
mild iodine deficiency vs. more than adequate iodine	1.61	-0.41–3.62	0.1188	0.04	
Region excessive iodine vs. more than adequate iodine	1.92	0.12–3.73	0.0369	0.05	

^a^: Sex was fixed in the model.

## Discussion

To our knowledge, this 5-year prospective study conducted in the three different (mild deficient, more than adequate, and excessive) iodine intake regions was the first attempt in China to determine the effect of iodine intake on serum Tg in adults. Our results demonstrated that the serum levels of Tg in both mild deficient and excessive iodine intake regions were substantially higher than that in more than adequate iodine intake region. As expected, the serum levels of Tg were substantially increased with age in all iodine intake regions. Furthermore, ΔTg appeared to be higher in the mildly deficient iodine intake region and became the greatest in the excessive iodine intake region, which indicates the time-accumulative effect of iodine exposure on Tg in adults: the more iodine intake, the greater ΔTg.

**Fig 1:**
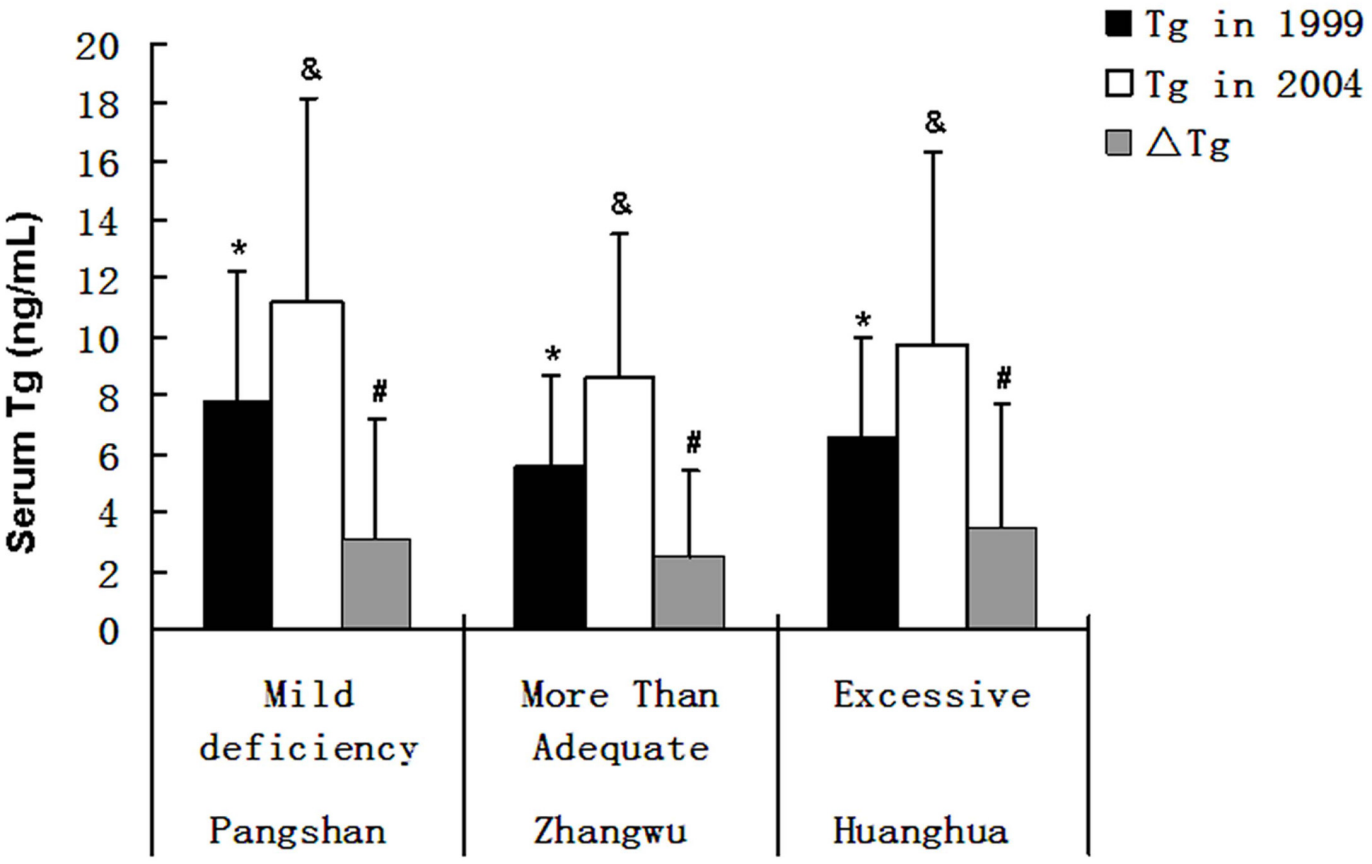
Serum Tg (ng/mL) in 1999,2004 and the 5-year changes of serum Tg (ΔTg) among different iodine status (n = 1856). 1856 subjects who had negative TgAb, normal TV and TSH, and negative personal history of thyroid diseases were from three levels of iodine status. Data were expressed as median + 1/2 interquartile range. * denotes a substancial difference among three levels of iodine status in 1999 (P<0.0001). ^&^ denotes a substancial difference among three levels of iodine status in 2004 (P<0.0001). ^#^ denotes a substancial difference of 5-year changes of Tg among three levels of iodine status (P = 0.0021).

Among the study population at baseline, all the evaluated factors, including age, gender, the presence of positive TgAb, goiter, abnormal TSH, and positive personal history of thyroid diseases, affected serum Tg substantially, which is accordant with the Laboratory Medicine Practice Guidelines [16]. In order to control the effects of these confounding factors, the data from subjects who had negative TgAb, normal TV, normal TSH, and negative personal history of thyroid diseases were analyzed to determine the effect of iodine intake on serum Tg. In both cross-sectional and prospective investigations, we have demonstrated that iodine intake was an independent determinant of serum Tg in adults. Both iodine deficiency and excessive iodine intake status had a positive effect on serum Tg in adults, and their dose-effect relationship tended to be close to a U-shaped curve (Fig 1). Zimmermann et al had reported similar results in school children [9].

Besides iodine intake, age was also found to be positively associated with serum Tg in this study, consistent with the results of a cross-sectional study [11]. This 5-year cohort study indicated that the serum levels of Tg were substantially higher in 2004 than those in 1999 in all three regions. After the adjustment of other related factors, age was still substantially associated with ΔTg. All these findings strongly suggested that age was an independent determinant of ΔTg. However, the contribution of region and age to the model R-Square accounted for 11.7% and 7.8% respectively, which indicated iodine intake affected ΔTg more than age did.

In our baseline study population, we found that the serum Tg in women was substantially higher than that in men. Similar observation had been reported by Knudsen et al [11]. This finding might be attributed to the effects of estrogen [17]. However, our 5-year follow-up results did not show any difference in ΔTg between females and males, which increased the likelihood that, even if the absolute serum Tg level differed between women and men, there is no gender difference in the change of Tg with aging.

Abnormal TSH substantially affected serum Tg in the present cohort study. Moreover, the baseline TSH level, even within the normal range, was still an independent determinant of ΔTg. Thus TSH affected both the levels of serum Tg and the magnitude of its change (Fig 1). This fact further suggested that serum Tg is sensitive to TSH stimulation. In addition, the general linear model analysis showed that the Tg level at baseline was another independent determinant of ΔTg. Actually, Tg level at baseline itself reflected the time-accumulative effect of iodine intake and TSH.

One limitation of the present study population was sex composition. Actually the problem of the sex imbalance (male:female≈1:3) occurred in all the three regions. This phenomenon was due to the mainstream lifestyle in rural areas in China: men leave home to work in cities, and stay there most time of the year; whereas women stay at home farming and taking care of the children and the elderly. Despite this imbalance, neither the distribution of gender was substantially different among the three regions nor was the present analysis interfered. Thus, the sex imbalance seemed unlikely to affect our conclusions.

After the effects of other related factors were controlled, iodine intake level was proved to substantially affect serum Tg level in adults. Serum Tg level in adults could be a sensitive biomarker of the regional iodine status. When serum Tg in healthy adults is employed to monitor the regional iodine exposure level, age should be controlled at the same time.

In keeping with the study reported by Zimmermann et al [9], we showed that the Tg level in subjects with more than adequate iodine status was around the bottom of a U-shaped curve. If the median Tg level increases in age-controlled healthy adults in a given population, this suggests the iodine status is out of the adequate range, either deficient or excessive. These two conditions can be identified via assessing median urinary iodine concentration, and the causes of iodine deficiency or excessiveness can be identified via checking salt iodine content at the production, importation, or household levels. On the contrary, if the median Tg level decreases, this means the iodine status tends to recover to or maintain within the safe level.

A mandatory universal salt iodization (USI) program was introduced nationwide in 1996 in China, with a recommended standard for the concentration of iodine in iodized salt. National standards for iodized salt in China had been revised twice to reduce the salt iodine concentration in 2002 and 2012 respectively, because the national data of median urinary iodine concentration infers that the Chinese population has been exposed to excessive iodine intake for 6 years and to more than adequate iodine intake for 11 years since the introduction of USI [18]. The results of the present population-based cohort study (1999–2004), especially the Tg levels obtained in 2004, are able to provide the baseline data for the latest revision of national standards for iodized salt in China in 2012. Iodine supplementation should be implemented to prevent and treat IDDs. However, iodine intake must be maintained at a safe level, because more than adequate or excessive iodine levels are unsafe and may lead to hypothyroidism and autoimmune thyroiditis, especially in susceptible populations with recurring thyroid disease, the elderly, fetuses, and neonates [18]. Iodine status of population should be closely monitored via the median urinary iodine concentration and median Tg in adults.
